# Social support and intimate partner violence during pregnancy among women attending antenatal care in Moshi Municipality, Northern Tanzania

**DOI:** 10.1186/s12889-017-4157-3

**Published:** 2017-03-09

**Authors:** Geofrey Nimrod Sigalla, Vibeke Rasch, Tine Gammeltoft, Dan Wolf Meyrowitsch, Jane Rogathi, Rachel Manongi, Declare Mushi

**Affiliations:** 1 Institute of Public Health, Kilimanjaro Christian Medical University College, Moshi, Tanzania; 2 Department of Health, Evangelical Lutheran Church in Tanzania, Arusha, Tanzania; 3 Department of Obstetrics and Gynecology, University of Southern Denmark, Odense, Denmark; 4 Department of Anthropology, University of Copenhagen, Copenhagen, Denmark; 5 Department of Public Health, University of Copenhagen, Copenhagen, Denmark; 6 Department of Community Health, Kilimanjaro Christian Medical Center, Moshi, Tanzania

**Keywords:** Intimate partner violence, Pregnancy, Social support, Tanzania

## Abstract

**Background:**

Intimate Partner Violence (IPV) is a significant public health problem with negative health consequences for women and their pregnancies. While social support has a protective effect against IPV and reduces health consequences of violence, its association with experiencing IPV during pregnancy remain less explored. In our study we aimed to determine the effect of social support on IPV during pregnancy among women attending antenatal care in Moshi, Tanzania

**Methods:**

The study was part of a prospective cohort study that assessed the impact of violence on reproductive health of 1,116 participants. Pregnant women were enrolled below 24 weeks of gestation and followed until delivery. The experiences of social support and IPV during pregnancy were assessed at the 34^th^ week of gestation. Logistic regression analysis was performed to assess the relationship between social support and IPV, with adjustment for potential confounders.

**Results:**

The prevalence of IPV during pregnancy was 30.3% where the majority (29.0%) experienced repeated episodes of abuse. Regarding practical social support, having no one to help financially was associated with increased odds of IPV and repeated episodes of abuse during pregnancy, AOR 3.57, (95% CI 1.85 - 6.90) and AOR 3.21, (95% CI 1.69 - 6.11) respectively. For social support in terms of communication, talking to a member of the family of origin at least monthly was associated with decreased odds of IPV and repeated episodes of IPV during pregnancy, AOR 0.46 (95% CI 0.26 - 0.82) and AOR 0.41 (95% CI 0.23 - 0.73) respectively. Perceiving that family of origin will not offer support was associated with a increased odds of IPV and repeated episodes of IPV, AOR 2.29, (95% CI 1.31 – 3.99) and AOR 2.14, (95% CI 1.23 – 3.74) respectively.

**Conclusions:**

Nearly one third of women experienced IPV during pregnancy. Social support to women is associated with decreased odds of experiencing IPV during pregnancy. The family of origin plays an important role in providing social support to women who experience abuse during pregnancy; however, their true involvement in mitigating the impact of violence in the African setting needs further research.

## Background

Violence against women is recognized as a significant public health problem that relates to gross violation of women’s human rights, affecting millions of women worldwide [1, 2]. Reports show that a woman is more likely to be hit, assaulted or murdered by her intimate partner than by a stranger, placing Intimate Partner Violence (IPV) as the most pervasive form of violence [2, 3]. Globally, one in three women are reported to have experienced IPV in their lifetime, with higher estimates documented in African countries [2], where prevalence rates range from 28 to 37% [4–6]. In Tanzania, four in ten women experienced IPV in their lifetime [7, 8].

Focusing on experiences of IPV during pregnancy, a review of African studies indicated that the prevalence of violence is one of the highest reported globally and ranged from 2 to 57% [9]. Studies have indicated that IPV during pregnancy affects health of women and pregnancy [10–12]. Peterson et al. conceptualized the effects of IPV to pregnancy and indicated that physical violence may cause direct injury to the gravid uterus leading to adverse pregnancy outcomes [13]. Alternatively, trauma and stress may indirectly affect the pregnancy through influencing negatively the health seeking behavior, precipitate women prenatal risks such as alcohol and substance abuse. A recent study done among Vietnamese women has shown that IPV during pregnancy is associated with preterm delivery (below 37 weeks of gestation) and low birth weight (less than 2,500 g) [10]. Preterm and low birth weight delivery impairs neonatal health leading to increased morbidity and mortality [14]. In Tanzania, preterm delivery and low birth weight constitute significant public health problems where they are responsible for up to 80% of all neonatal deaths and one-third of all deaths among children under-five years of age [15]. IPV during pregnancy have also been associated with pregnancy loss, miscarriage and stillbirth [11, 12].

Due to the negative health consequences of IPV during pregnancy, previous research has focused on a general assessment of factors associated with experiencing violence among pregnant women so as to aid efforts in the prevention of IPV and mitigate its health consequences [9, 12, 16–19]. There is evidence documenting risk factors associated with experiencing violence, which include young age [9], alcohol use by women [12] and their partner [18], high parity [19] and previous history of adverse pregnancy outcomes [16].

One potential strategy for mitigating exposure to IPV during pregnancy is social support. In that regard, there is a growing focus on understanding the association between social support and IPV in order to inform future interventions to prevent IPV and reduce the resulting complications of violence, especially during pregnancy. Social support is defined as the assistance women receive from other people and through supportive social networks regardless of whether the support is merely expected (perceived) or actually received by the beneficiary [20]. Social support may be grouped into five broad categories; emotional social support (advice, feedback), communication with family members, perceived social support from family members, group social support and practical social support (tangible help such as food, money and pregnancy care). The advantages of social support to maternal and fetal wellbeing are known [21–23]. Women who receive social support are less likely to report depressive symptoms during pregnancy [21] irrespective of educational level, wealth status, occupation, perceived work burden, food security, history of miscarriage or stillbirth and whether the pregnancy was planned or not. Dibaba et al. argue that social support during pregnancy plays a “buffering role” from depression [21]. Women who reported being satisfied with social support during pregnancy had babies born with higher birth weights [22]. On the other hand, women who lacked adequate social support had a higher risk of pregnancy complications such as miscarriage, pre-eclampsia and preterm births [23]. It may be concluded that social support during pregnancy is an important factor for maternal and fetal wellbeing.

The association between social support and partner violence has been described in the general population of women [24–26] but there is only limited knowledge regarding the association between social support and IPV during pregnancy [27]. Social support has been known to be a protective factor for women who are exposed to abuse by their partner [20, 23] and reduces the health consequences of violence [21, 22]. Studying 500 women in Pakistan, Farid et al. found that women with social support were less likely to experience abuse from their partner [27]. Wright et al. later showed that women who had social support from members of their family had reduced prevalence and frequency of IPV [24]. Social support mediates the relationship between abuse and distress, leading to lower levels of negative psychological effects [28], a mechanism responsible for positively influencing the negative health consequences that result from violence. In sub-Saharan Africa, Tanzania included, the majority of women who experience IPV find family members and friends to be their primary contacts when compared to formal institutions like police and legal aid [29–31]. Tanzanian women make use of informal social support networks for maternal and child care [32] but it remains unclear whether such networks influence the risk of exposure to IPV during pregnancy.

The need for strong evidence on the association between social support and experiencing IPV during pregnancy is necessary for designing future interventions to prevent violence against women during pregnancy. The aim of this study was therefore to determine the association between social support and IPV during pregnancy among women attending antenatal care in Moshi Municipality, northern Tanzania.

## Methods

### Study design and settings

This study was nested within a larger cohort study conducted among pregnant women attending antenatal care (ANC) before the 24^th^ gestational week in Moshi Municipality, Tanzania, and used a cross-sectional study design. To limit the time burden to participants, the interviews were divided into three time periods; at enrolment where socio-demographic and reproductive health information were collected, at 34 weeks of gestation where exposure to IPV before and during pregnancy was assessed, and within 48 h post-delivery where gestation age at delivery and birth weight were determined.

The study was conducted at Majengo and Pasua Health Centers in Moshi Municipality, Kilimanjaro Region, Tanzania. The two clinics are located in the semi-urban areas of Moshi Municipality. There are 23 clinics in Moshi Municipality that offer primary ANC services to about 7,000 – 8,000 pregnant women annually. Nearly half of all the pregnant women in the Municipality receive ANC services at Majengo and Pasua Health Centers. About the Municipality, it is one of the seven districts of Kilimanjaro Region and with estimated population of 206,728 people. Located on the slopes of Mount Kilimanjaro, a snow-capped and the highest mountain in Africa, the municipality was once famous for its robust economy from coffee. The falling prices of coffee in the international market forced the residents of the municipality to start small scale farming of crops such as maize and banana. Apart from subsistence farming, women in the area do engage in selling agricultural produce to the market. Women have also opened small shops which are locally called *‘kiosk’* and sell either clothes or items for household use such as soap, sugar, salt and soft drinks.

### Participants, recruitment and data collection

The study population included women registered at the two clinics for antenatal care between March 2014 and May 2015. Inclusion criteria were: pregnant women aged 18 years or above, who were planning to deliver within Moshi Municipality and with pregnancy gestational age of less than 24 weeks as confirmed by ultrasound scan. Exclusion criteria were: not living in Moshi Municipality, not willing to be followed up for the entire period of study and having multiple pregnancies.

The research assistants comprised six female nurses, aged above 35 years, who were experienced in research and committed fulltime for the entire period of the research. Research assistants were trained for five days on how to conduct this study with regard to its sensitive nature. The enrolment and follow-up interviews were conducted in a private room at the clinic where no one other than the research assistant and the participant were allowed to be present, except children under two years of age. All information was collected through face-to-face interviews in *Swahili* language. The standard enrolment interview questionnaire included information on socio-demographic and reproductive health characteristics. The follow-up interview assessed social support and exposure to IPV before and/or during pregnancy. The two interviews each lasted between 45 and 60 min.

## Measures

### Independent variables

#### Demographic and reproductive health characteristics

These were age (in years), highest level of education attained (never attended school, primary, secondary and above secondary) and occupation (employed by government or private organization, self-employed as farmer or business, unemployed). Reproductive health characteristics assessed were “number of pregnancies”, “if the current pregnancy was planned (yes or no)” and “any previous history of adverse pregnancy outcomes such as miscarriage, stillbirth and previous delivery of a premature or low birth weight baby (yes or no to any)”. Assessment of alcohol use during current pregnancy (yes or no) as a health risk behavior was also undertaken.

#### Social Support

Three questions assessed emotional forms of social support: if the participant had “someone to share her own thoughts and worries”, “someone to help in making difficult decisions” and/or “someone to always trust”. Communication with family of origin and of the partner was assessed using two questions; “how often the participant talked with a member of her family of origin and a member of the partner’s family (at least once a week, a month or a year)” based on the highest frequency of communication. Perceived support was assessed using two questions: “whether, in case of problems, the participant would count on a member of her family of origin or a member of the partner’s family for support (yes or no)”. One question on group support was: “whether the participant was attending any women’s or community groups, religious or political groups (yes or no)”. Four questions assessed practical social support, where each participant was asked if there was someone who “cares by making sure she gets enough to eat”; “someone who helps with daily tasks”; “someone with positive interest that she attends the ANC clinic”; and “someone to depend on financially when in need”.

### Dependent variable

Intimate partner violence was assessed using a *Swahili* version of the tool previously used in Tanzania in the WHO Multi-Country Study on Women’s Health and Domestic Violence against Women [8]. The assessed periods were “ever” and “during pregnancy”. Physical Violence was defined based on six questions; a woman reporting to have been either “slapped or have had something thrown at her that could hurt”, “pushed or shoved or pulled her hair”, “hit with a fist or something else that could hurt”, “kicked or dragged or beaten”, “choked or burnt on purpose” and/or been “threatened with a gun, knife and other weapon, or actual use of these, against her” by her partner. Acts that defined sexual violence were based on three questions: being “physically forced by her partner to have sexual intercourse when she did not want to”, “had sexual intercourse she did not want because she was afraid of what her partner might do if she refused” and/or “ever forced to do something sexual that she found degrading or humiliating”. Emotional violence was defined based on four questions, such as when her partner “insulted her or made her feel bad about herself”, “belittled or humiliated her in front of other people”, had “done things to scare or intimidate her on purpose” and/or “threatened to hurt her or someone that she cares about”. Participants who reported to have ever experienced any act of violence were then asked two follow-up questions about timing - whether such experience occurred before or during the current pregnancy and the frequency, once, a few times (2–5 episodes) or many times (more than 5 episodes). The two dependent variables were: (1) experiencing any type of emotional, physical and/or sexual violence during pregnancy and (2) experiencing repeated episodes of emotional, physical and/or sexual violence during pregnancy.

#### Statistical methods

Responses obtained from participants were coded, double entered in EpiData v2.0.3.15, cleaned and later exported to the Statistical Package for Social Studies (SPSS) program version 20.0 for analysis. Social support variables were dichotomized as yes (always, most of the time and some of the time) or no (rarely or never). Responses on experience of emotional, sexual and physical violence were dichotomized as yes (if responded yes to one or more of the acts specified for the type of violence) and no (if responded no to all acts in the group). The ‘any violence’ variable was generated and categorized as yes (if she experienced any emotional, sexual or physical violence) and no (if experienced none). Frequency of experiencing violence during pregnancy was dichotomized as: experienced a single episode and those who experienced repeated episodes of violence (two or more episodes).

Descriptive statistics were used to summarize lifetime prevalence of violence, exposure to violence during pregnancy and frequency of experiencing episodes of violence during pregnancy. The socio-demographic and reproductive health characteristics of women were described by experiences of emotional, sexual, physical and/or any violence during pregnancy using the chi-square test to determine the difference between the groups. Bivariate logistic regression analysis was used to calculate the crude odds ratio (OR) with 95% confidence intervals for the association between social support factors and the two dependent variables. Multivariate logistic regression was used to examine the associations between social support variables that were statistically significant predictors of experiencing any type of IPV and experiencing repeated episodes of IPV during pregnancy in the bivariate logistic regression analysis. Confounding factors such as maternal age, level of education, occupation, unplanned pregnancy, history of previous adverse pregnancy outcome and alcohol consumption during pregnancy are known to be associated with violence and/or social support and were thus included in the final model of analysis to assess the effect of social support on violence exposure independent of these confounders.

## Results

A total of 1,123 pregnant women were enrolled. Seven women did not come for IPV assessment at week 34 and were excluded from the analysis, leaving a total of 1,116 participants. The mean age of all participants was 26 years (Standard deviation of 5.8 years) ranging between 18 and 44 years, with more than three quarters (79.3%) being aged between 20 and 35 years. Nine out of ten participants (89.8%) were living with their partners.

### Prevalence of IPV

Table 1 presents the prevalence of the three forms of violence assessed in this study. The lifetime prevalence of emotional violence was 30.8%, and 22.8% of all women had experienced emotional violence during their current pregnancy. The reported lifetime prevalence of physical violence was 10.7%, and 70 (6.3%) women had experienced physical violence during the current pregnancy. Finally, the lifetime prevalence of sexual violence was reported to be 19.3%, and 171 (15.3%) women had experienced sexual violence during the current pregnancy. Overall, 438 (39.2%) women reported having experienced at least one type of violence from an intimate partner in their lifetime while 30.2% had experienced it during the current pregnancy. About 29.0% of all women who experienced IPV during pregnancy reported having been exposed to two or more episodes of IPV during their pregnancy.

**Table 1:** Prevalence and 95% confidence intervals of various forms of violence (*n* = 1,116)

Intimate partner violence	Number of women who experienced specific type of IPV	Prevalence in % (95% CI)
Lifetime experience of violence		
Emotional violence	344	30.8 (28.0 – 33.7)
Physical violence	119	10.7 (9.0 – 12.6)
Sexual violence	215	19.3 (16.8 – 21.8)
At least one type of violence	438	39.2 (36.4 – 42.2)
Violence during pregnancy		
Emotional violence	254	22.8 (20.2 – 25.4)
Physical violence	70	6.3 (4.8 – 7.6)
Sexual violence	171	15.3 (13.3 – 17.6)
Emotional, physical or sexual violence	337	30.2 (27.4 – 32.9)
Repeated episodes of emotional violence^a^	242	21.7 (19.3 – 24.1)
Repeated episodes of physical violence^a^	62	5.6 (4.2 – 6.9)
Repeated episodes of sexual violence^a^	164	14.7 (12.7 – 16.8)
Repeated episodes of at least one type of violence^a^	324	29.0 (26.4 – 31.5)

^a^Experienced two or more episodes of abuse during the current pregnancy

Demographic and reproductive health characteristics of participants are presented in Table 2. The majority of participants 676 (60.6%) had attained primary school level education. About 512 (45.9%) participants were self-employed in petty business, selling agricultural produce and items for household use to the nearby markets. Among the participants who had ever been pregnant, a quarter (24.6%) reported a previous history of pregnancy that ended as miscarriage, stillbirth, preterm birth and/or low birth weight baby. Nearly a quarter (23.9%) of all participants reported that their current pregnancies were unplanned and one in ten (11.0%) had consumed alcohol during pregnancy.

**Table 2:** Demographic and reproductive health characteristics of the study participants, and the prevalence of exposure to IPV during pregnancy (*n* = 1,116, if no other indication)

Characteristic	No. of women (% of total)	Emotional violence		Physical violence		Sexual violence		At least one type of violence	
		No. of women (prevalence in %)	*P* value^a^	No. of women (prevalence in %)	*P* value^a^	No. of women (prevalence in %)	*P* value^a^	No. of women (prevalence in %)	*P* value^a^
Age (in years)									
18–20	144 (12.9)	30 (11.8)	0.486	7 (10.0)	0.828	18 (10.5)	0.238	39 (11.6)	0.502
21–25	374 (33.5)	79 (31.1)		22 (31.4)		53 (31.0)		105 (31.1)	
26–30	310 (27.8)	82 (32.3)		19 (27.1)		44 (25.7)		102 (30.3)	
31–35	167 (15.0)	36 (14.2)		13 (18.6)		33 (19.3)		50 (14.8)	
36 and above	121 (10.8)	27 (10.6)		9 (12.9)		23 (13.5)		41 (12.2)	
Education level completed									
Primary	676 (60.6)	149 (58.7)	0.737	44 (62.9)	0.771	108 (63.2)	0.575	207 (61.4)	0.871
Secondary	390 (34.9)	92 (36.2)		24 (34.3)		54 (31.6)		114 (33.8)	
Above secondary	50 (4.5)	13 (5.1)		2 (2.8)		9 (5.2)		16 (4.8)	
Occupation									
Employed	179 (16.0)	40 (15.7)	0.026	10 (14.3)	0.345	28 (16.4)	0.189	56 (16.6)	0.009
Self-employed	512 (45.9)	134 (52.8)		38 (54.3)		88 (51.5)		175 (51.9)	
Unemployed	425 (38.1)	80 (31.5)		22 (31.4)		55 (32.1)		106 (31.5)	
Parity									
0	429 (38.4)	83 (32.7)	0.072	18 (25.7)	0.053	63 (36.8)	0.892	115 (34.1)	0.068
1–2	533 (47.8)	129 (50.8)		38 (54.3)		84 (49.1)		166 (49.3)	
3 or more	154 (13.8)	42 (16.5)		14 (20.0)		24 (14.1)		56 (16.6)	
Index pregnancy planned?									
Yes	849 (76.1)	179 (70.5)	0.017	54 (77.1)	0.826	129 (75.4)	0.846	247 (73.3)	0.169
No	267 (23.9)	75 (29.5)		16 (22.9)		42 (24.6)		90 (26.7)	
Any history of adverse pregnancy outcome^b^ (*n* = 674)									
No	166 (24.6)	124 (73.4)	0.536	35 (67.3)	0.180	70 (67.3)	0.047	151 (69.3)	0.011
Yes	508 (75.4)	45 (26.6)		17 (32.7)		34 (32.7)		67 (30.7)	
Any alcohol consumption during pregnancy									
Yes	123 (11.0)	42 (16.5)	0.001	22 (31.4)	<0.001	26 (15.2)	0.058	56 (16.6)	<0.001
No	993 (89.0)	212 (83.5)		48 (68.6)		145 (84.8)		281 (83.4)	

^a^Results of chi-square test of statistical difference on the proportional distribution of exposure to violence

^b^Women who had a previous pregnancy that ended as miscarriage, stillbirth, preterm birth and/or low birth weight baby

Exposure to emotional violence was significantly more likely to occur among women who were self-employed, had an unplanned pregnancy and who consumed alcohol during pregnancy (Table 2). Exposure to physical violence was more likely among women who consumed alcohol during pregnancy while sexual violence was significantly more common among women who had a previous pregnancy that ended as miscarriage, stillbirth, preterm birth and/or low birth weight baby. Experiencing any type of violence during pregnancy was significantly more often reported among women who were self-employed, with a history of previous adverse birth outcome and those who consumed alcohol during pregnancy.

### Social support and experiencing IPV during pregnancy

Women who reported talking to a member of their family of origin and/or family of their partner at least once a month and those who indicated that they could count on support from their family of origin or their partner’s family had a decreased odds of experiencing IPV during pregnancy (Table 3). However, women who stated that they had no one who took interest in their ANC services, no one who helped them financially when needed, no one they could share thoughts and worries with, no one who would help them in decision making or no one they felt they could trust had increased odds of experiencing violence. Adjustment was performed to control for the influence of maternal age, level of education, occupation, unplanned pregnancy, history of adverse pregnancy outcome and any alcohol consumption during pregnancy (model 2). In the adjusted analysis, women who were talking to a member of their family of origin at least once monthly had significantly increased odds of experiencing any type of violence during pregnancy as compared to those who were talking to the family at least once weekly, AOR 0.46 (95% CI 0.26 - 0.82). Women who perceived that a member of the family of origin would not offer support when needed had significantly increased odds of experience any form of violence during pregnancy as compared to women who did expect any support from their family, AOR 2.29 (95% CI1.31 – 3.99). Likewise, women who reported having no one to depend on financially were at significantly increased odds of experiencing any type of violence during pregnancy as compared to those who had someone to depend on financially, AOR 3.57 (95% CI 1.85 – 6.90).

**Table 3:** Association between women social support factors and exposure to at least one type of IPV during pregnancy (*n* = 1,116, if no any other indication)

Variable	No. of women (% of total)	Exposure to at least one type of IPV (prevalence in %)	OR (95% CI)	Model 1^a^ Adj. OR (95% CI)	Model 2^b^ Adj. OR (95% CI)
Talking to family					
Talks to family of origin					
At least once a week	575 (51.5)	190 (33.0)	1.00		
At least once a month	340 (30.5)	74 (21.8)	0.49 (0.33 – 0.72)	0.55 (0.38 - 0.80)	0.46 (0.26 – 0.82)
Once a year or never	201 (18.0)	73 (36.3)	0.87 (0.62 – 1.21)	-	-
Talks to family of the partner					
At least once a week	522 (46.8)	163 (31.2)	1.0		
At least once a month	349 (31.3)	82 (23.5)	0.51 (0.36 – 0.73)	0.75 (0.52 - 1.08)	**-**
Once a year or never	245 (22.0)	92 (37.6)	0.76 (0.55 – 1.04)	-	-
Perceived support from the family					
Expect support from family of origin					
Yes	943 (845)	257 (27.3)	1.0		
No	173 (15.5)	80 (46.2)	2.31 (1.66 – 3.21)	2.15 (1.39 – 3.32)	2.29 (1.31 – 3.99)
Expect support from family of the partner					
Yes	292 (26.2)	218 (26.5)	1.0		
No	824 (73.8)	119 (40.8)	1.91 (1.45 – 2.53)	1.64 (1.14 – 2.28)	1.21 (0.81 – 1.83)
Group support					
Attends association or organization					
Yes	160 (14.3)	57 (35.6)	1.0	-	-
No	956 (85.7)	280 (29.3)	0.75 (0.53 – 1.07)		
Practical support					
Has someone who cares that she gets enough food^c^					
Yes	888 (79.8)	259 (29.2)	1.0		
No	225 (20.2)	78 (34.7)	1.29 (0.95 - 1.76)	-	-
Has someone to help on daily tasks^c^					
Yes	649 (58.3)	200 (30.8)	1.0		
No	464 (41.7)	137 (29.5)	0.94 (0.73 - 1.22)	-	-
Has someone to support during ANC visits					
Yes	871 (78.3)	249 (28.6)	1.0		
No	242 (21.7)	88 (36.4)	1.43 (1.06 - 1.93)	0.90 (0.57 - 1.41)	-
Has someone to help her financially when in need^c^					
Yes	1037 (93.2)	290 (28.0)	1.0		
No	76 (6.8)	47 (61.8)	4.18 (2.58 - 6.76)	3.91 (2.05 - 7.46)	3.57 (1.85 – 6.90)
Emotional support					
Has someone to share thoughts and worries with^c^					
Yes	1037 (93.3)	299 (28.8)	1.0		
No	75 (6.7)	38 (50.7)	2.54 (1.58 - 4.07)	1.32 (0.60 - 2.91)	**-**
Has someone to assist when making difficult decisions^c^					
Yes	1036 (93.1)	301 (29.1)	1.0		
No	77 (6.9)	36 (46.8)	2.14 (1.34 - 3.42)	1.18 (0.42 - 3.32)	**-**
Has someone she can always trust^c^					
Yes	1049 (94.2)	308 (29.4)	1.0		
No	64 (5.7)	29 (45.3)	1.99 (1.20 - 3.32)	1.41 (0.48 - 4.13)	**-**

^a^Model 1 includes all factors with *p* value of <0.05 in the crude results

^b^Model 2 includes additional adjustments for maternal age, level of education, occupation, unplanned pregnancy, any history of adverse pregnancy outcome and any alcohol consumption during pregnancy

^c^Numbers do not add up to the total because of missing values

### Social support and experiencing repeated episodes of IPV during pregnancy

Women who reported talking to a member of their family of origin and/or family of their partners at least once a month and those who reported that they could count on support from family of origin or family of the partner had decreased odds of experiencing repeated episodes of IPV during pregnancy (Table 4). On the other hand, women who stated that they had no one who took interest in their ANC services, helped them financially when needed, they could share their thoughts and worries with, would help them in decision making or who indicated that they could trust had increased odds of experiencing repeated episodes of violence during pregnancy. The adjusted analysis (model 2) showed that women who talked to a member of their family of origin at least once monthly had significantly increased odds of experiencing repeated episodes of IPV as compared to those women who were talking to the family at least once weekly, AOR 0.41 (95% CI 0.23 - 0.73). Similarly, women who did not anticipate that a member of their family of origin would offer support when in need had significantly increased odds of experience repeated episodes of IPV as compared to women who did not expect any support from their family, AOR 2.14, (95% CI 1.23 – 3.74). An association between financial dependency and IPV was also found, hence women who reported having no one to depend on financially had a significantly increased odds of experiencing repeated episodes of IPV when compared to those who had someone to depend on financially, AOR 3.21 (95% CI 1.69 – 6.11).

**Table 4:** Association between women social support factors and experiencing repeated episodes of IPV during pregnancy (*n* = 1,116, if no any other indication)

Variable	No. of women (% of total)	Repeated episodes of violence^a^ (prevalence in %)	OR (95%CI)	Model 1^b^ Adj. OR (95% CI)	Model 2^c^ Adj. OR (95% CI)
Talking to family					
Talks to family of origin					
At least once a week	575 (51.1)	180 (31.3)	1.0		
At least once a month	340 (30.5)	71 (20.9)	0.46 (0.31 – 0.68)	0.55 (0.38 – 0.80)	0.41 (0.23 – 0.73)
Once a year or never	201 (18.0)	73 (36.3)	0.80 (0.57 – 1.12)	-	
Talks to family of the partner					
At least once a week	522 (46.8)	155 (29.7)	1.0		
At least once a month	349 (31.3)	80 (22.9)	0.52 (0.36 – 0.75)	0.80 (0.55 – 1.15)	-
Once a year or never	245 (22.0)	89 (36.3)	0.74 (0.54 – 1.02)	-	
Perceived support from the family					
Expects support from family of origin					
Yes	943 (84.4)	245 (26.0)	1.0		
No	173 (15.5)	78 (45.1)	2.34 (1.67 – 3.26)	2.14 (1.38 – 3.31)	2.14 (1.23 – 3.74)
Expects support from family of the partner					
Yes	292 (26.2)	212 (25.7)	1.0		-
No	824 (73.8)	112 (38.4)	1.80 (1.35 – 2.38)	1.47 (1.02 – 2.13)	1.12 (0.74 – 1.68)
Group support					
Attends association or organization					
Yes	160 (14.3)	54 (33.8)	1.0	-	-
No	956 (85.7)	270 (28.2)	1.29 (0.91 – 1.85)		
Practical support					
Has someone who cares that she gets enough food^d^					
Yes	888 (79.8)	248 (27.9)	1.0		
No	225 (20.2)	76 (33.8)	1.32 (0.96 – 1.80)	-	-
Has someone to help on daily tasks^d^					
Yes	649 (58.3)	192 (29.6)	1.0		
No	464 (41.7)	132 (28.4)	0.95 (0.73 – 1.23)	-	-
Has someone to support during ANC visits^d^					
Yes	871 (78.3)	239 (27.4)	1.0		
No	242 (21.7)	85 (35.1)	1.43 (1.06 – 1.94)	0.94 (0.60 – 1.48)	-
Has someone to help her financially when in need^d^					
Yes	1037 (93.2)	279 (26.9)	1.0		
No	76 (6.8)	45 (59.2)	3.94 (2.45 – 6.36)	3.52 (2.16 – 5.74)	3.21 (1.69 – 6.11)
Emotional support					
Has someone to share thoughts and worries with^d^					
Yes	1037 (93.3)	287 (27.7)	1.0		
No	75 (6.7)	37 (49.3)	2.54 (1.59 – 4.08)	1.08 (0.39 – 2.98)	-
Has someone to help in difficult decisions^d^					
Yes	1036 (93.1)	290 (28.0)	1.0		
No	77 (6.9)	34 (44.2)	2.03 (1.27 – 3.25)	0.99 (0.35 – 2.78)	-
Has someone she can always trust^d^					
Yes	1049 (94.2)	296 (28.2)	1.0		
No	64 (5.7)	28 (43.8)	1.98 (1.19 – 3.30)	1.31 (0.46 – 3.79)	-

^a^ Experienced two or more episodes of abuse during the current pregnancy

^b^Model 1 includes all factors with *p* value of <0.05 in the crude results

^c^Model 2 includes additional adjustment for maternal age, level of education, occupation, unplanned pregnancy, any history of adverse pregnancy outcome and any alcohol consumption during pregnancy

^d^Numbers do not add up to the total because of missing values

## Discussion

According to our knowledge, this is the first study to assess the association between social support and IPV during pregnancy in Tanzania. This study indicates that nearly four in ten women (39.2%) had ever experienced intimate partner violence, and close to one-third (30.2%) were exposed to IPV during pregnancy. Almost one-third of the women (29%) experienced repeated episodes of abuse during pregnancy. While women who had no one to depend on for financial help were at increased risk of experiencing IPV during pregnancy, those who were in communication with a member of their family of origin at least once a month and trusted that a member of the family would be there to offer support in case of need had decreased odds of experiencing IPV during pregnancy.

The results of this study show that acts of violence are a problem during pregnancy among Tanzanian women where three in ten women are exposed to IPV during pregnancy. These results are consistent with findings from a previous study done among pregnant women attending antenatal care in Dar es Salaam, Tanzania, where the prevalence of physical and sexual violence during pregnancy was 27% [11]. The results of the present study are also comparable with findings from other studies done among pregnant women attending antenatal care in neighboring countries [4–6]. While the prevalence of violence among 600 Ugandan women was 27.7% [4], Makayoto et al. reported the prevalence of IPV among 300 pregnant women attending antenatal care in Kenya to be 37% [5]. Comparable IPV prevalence of 35.1% among 600 pregnant women, assessed over a duration of 12 months that included the pregnancy period was reported in Rwanda [6].

Nearly all women who had experienced violence during pregnancy (97%) reported that they had experienced repeated episodes. This implies that violence during pregnancy is not a one-off event. It appears as violence is common and a part of women’s lives during pregnancy. In Zimbabwe, one in ten women reported having experienced six or more episodes of violence during pregnancy [33]. Although the negative health impacts of violence exposure may be assessed from the consequences of a single event, repetitive acts of violence are likely to be associated with higher health risk for the woman and the pregnancy.

The most important social support factors associated with IPV during pregnancy, and repeated episodes of violence, are the communication with and perceived support from members of the family of origin, and financial support. Although group support and emotional support are crucial inputs for pregnancy care, their association with experiencing violence during pregnancy was statistically insignificant.

Communication with a member of the family of origin and trusting that the family will offer support in case of problems has been found to be associated with decreased odds of exposure to IPV and decreased odds for exposure to repeated episodes of violence during pregnancy. This implies that the strong family ties and networks established between the woman and the family of origin are associated with decreased odds of exposure to violence during pregnancy.

The findings in our study, that women who communicate with a member of family of origin at least once a week were associated with increased odds of experiencing IPV during pregnancy than those who were talking at least once a month, were not expected and need to be discussed. While further exploratory research is needed to understand this association, we will try to suggest a plausible explanation. Women who are in a stressful state as a result of violence will likely be in dire need of emotional support. These women will likely disclose their relationship challenges to the family of origin. In turn, they are motivated to continue staying in their relationship. Such a sharing between the women and their family of origin would increase the frequency of communication. In other words, violence may change the communication habits of those exposed to violence. However, there is complexity around the association between communication and social support. This study provided no information about the nature of the contacts and whether such contacts were supportive or not. In that case, the frequency of communication with the family of origin may not be equivalent to social support, implying that the frequency of contact is not a good variable for assessing social support.

The results of our study are comparable to the findings from a large national representative survey in Turkey on what puts women at risk of violence from their husbands. The study showed that women who are close to the family of origin are likely to receive emotional and/or physical support from them, a role that in itself is associated with reduced risk of experiencing violence [34]. Yuksel-Kaptanoglu et al. further showed that preventing women from contacting their family was associated with increased risk of experiencing violence. Underscoring the role of family of origin in violence, an additional study in Turkey found that women do prefer to disclose their experience of IPV to the family of origin compared to the family of the partner even though they may not receive any type of support from them [35]. In the same study however, the support from the family of their partner was found to be associated with increased risk of violence and highlighted the contradicting role of families to the woman. It may therefore be noted that the response of the partners’ family is not predictable when it comes to a point of choosing who to support when conflict arise: their son or the woman who is abused. In Tanzanian culture, especially when the couple lives close to the family of the partner, conflicts may result when mothers-in-law exercise control and power in issues of space, food, finances and decisions. This complexity underscores the fact that IPV is part of gender based violence and therefore understanding the context in which it happens [36] require another level of conceptualization of how family structures and patriarchal ideology affect women’s lives.

Of all actions that constituted practical support for women during pregnancy, the findings of this study confirm what has been documented in other studies, namely that economic dependency increases the risk of IPV [37–39]. Unraveling the complex relationship between dependency and domestic violence, Schewe hypothesized that when there is dependence on others for financial assistance there is a possibility that the dependent member may be mistreated or exploited, regardless of the source of support [37]. Focusing on IPV and studying 1,886 women from national representative data on IPV in USA, Golden et al. found that women who depended on their intimate partner economically were at increased risk of one or more types of IPV regardless of the women’s’ race or ethnic origin [38]. Bornstein et al. showed that it is not only women’s economic dependency on men that leads to domestic violence, but also their emotional dependency [39]. Women therefore become more vulnerable to IPV when they have to depend on their intimate partner economically and emotionally. Women’s dependency to their partners economically especially during pregnancy period may explain as to why in our study self-employed women were more likely to report IPV. In case of health related complications of pregnancy, some women may work less both at home and/or in other activities outside home, leading to decreased income that may have otherwise gained if she was not pregnant. Especially for self employed women in business, some of them are out of their job during their pregnancy period. They are then transiently or permanently economically dependent to their intimate partner increasing their vulnerability to IPV.

In many African settings, men are responsible for providing financial support to the family. The child to be born will create an economic challenge to the family, which is probably already in a situation of financial crisis, posing a new demand to care for the child and mother after delivery [40]. The increasing number of children may therefore present uncertainties to the partner in terms of financial support to the family during pregnancy and after delivery [41]. Bacchus et al. further found that men’s doubts about parenting of their awaited child increased physical and emotional violence for women during pregnancy. Women's constant requests for support may also increase frequency of abuse, especially when the intimate partners cannot support the woman economically. While financial dependency by itself was associated with experiences of repeated episodes of violence, it presents uncertainties in relation to women’s future support and may limit the way they will respond to prevent further violence.

### Strengths and limitations of the study

This study has some limitations that should be considered in interpreting of the results. The cross-sectional nature of the study makes it 'impossible to draw causal inferences, preventing establishment of the direction of causality between social support and IPV during pregnancy. Experiences of intimate partner violence may be under-reported, considering the cultural sensitivity around issues of violence, leading to under-estimation of the strength of the association between social support and IPV; this could be a factor despite the fact that the research assistants interviewed women in a non-judgmental way after they established good rapport. Moreover, women who did fit the inclusion criteria of below 24 weeks pregnancy gestation may have different characteristics to those who register late at antenatal clinics for service, limiting generalization of the results. However, data show that at least two-thirds of women in the area of study register for their first antenatal visit before the 24^th^ week of their pregnancy and nearly all women attend antenatal care at least once over the entire period of their pregnancy [7]. Although some measures of social support (communication, perceived support and group support) included time period before pregnancy, we may not ascertain whether there was change in social support received by women because of pregnancy. Also, this study did not measure the level of family income or poverty. However, the study recruited relatively a large sample size of pregnant women and used validated tools for assessment of violence. The use of validated tools ensures comparability with results from other studies done elsewhere.

## Conclusions

The study found that three in ten women experienced at least one type of IPV during the index pregnancy and most had experienced repeated episodes of violence. Women’s financial dependency was associated with increased odds of experiencing violence during pregnancy. Close ties with family of origin and trust in receiving support from them in case of problems were associated with decreased odds of violence during pregnancy. Reducing women’s financial dependency through economic empowerment to establish and/or expand their business so as to sustain them during the pregnancy and post partum period might reduce their vulnerability to IPV. Focusing on non-formal support networks, a review of interventions on social support which was done by Small et al. showed that what matters most to women is the sense of companionship that entail “not feeling so alone, being understood, not being judged and increased sense of own worth” [42]. In African setting where members of the family of origin and family of the partner are part of important community networks of women during pregnancy, targeted interventions to constructively engage them in providing social support will benefit women victims of partner violence. Women attendance to antenatal clinics offers golden opportunity to be explored in engaging these informal networks when they accompany women to points of heath care. Such engagement in provision of social support during pregnancy will benefit the pregnancy and the prevention and mitigation of impact of IPV. The role of family of origin in preventing IPV should be explored further so that family members are correctly positioned on how to support the abused, taking into consideration the context of African settings where violence occurs. Interventions aiming at addressing gender based violence may find information from this study useful in programming for social support during pregnancy.

## Abbreviations

95% CI: 95% confidence interval
ANC: Antenatal Care
AOR: Adjusted odds ratio
IPV: Intimate partner violence
OR: Odds ratio
RERC: Research and Ethical Review Committee
SPSS: Statistical Package for Social Sciences
WHO: World Health Organization
